# *In Vitro* Characterization of Protein Effector Export in the Bradyzoite Stage of Toxoplasma gondii

**DOI:** 10.1128/mBio.00046-20

**Published:** 2020-03-10

**Authors:** Joshua Mayoral, Peter Shamamian, Louis M. Weiss

**Affiliations:** a Department of Pathology, Albert Einstein College of Medicine, New York, New York, USA; b Department of Medicine, Albert Einstein College of Medicine, New York, New York, USA; Stanford University

**Keywords:** GRA proteins, TgIST, *Toxoplasma gondii*, bradyzoite, effector functions, interferon gamma

## Abstract

The ubiquitous parasite Toxoplasma gondii exhibits an impressive ability to maintain chronic infection of its host for prolonged periods. Despite this, little is known regarding whether and how T. gondii bradyzoites, a quasi-dormant life stage residing within intracellular cysts, manipulate the host cell to maintain persistent infection. A previous proteomic study of the cyst wall, an amorphous layer of proteins that forms underneath the cyst membrane, identified MYR1 as a putative cyst wall protein *in vitro*. Because MYR1 is known to be involved in the translocation of parasite-derived effector proteins into the host cell, we sought to determine whether parasites transitioning toward the bradyzoite life stage retain the capacity to translocate proteins via this pathway. By epitope tagging the endogenous loci of four known effectors that translocate from the parasitophorous vacuole into the host cell nucleus, we show, by immunofluorescence assays, that most effectors accumulate in the host nucleus at early but not late time points after infection, during the tachyzoite-to-bradyzoite transition and when parasites further along the bradyzoite differentiation continuum invade a new host cell. We demonstrate that the suppression of interferon gamma signaling, which was previously shown to be mediated by the effector TgIST, also occurs in the context of prolonged infection with bradyzoites and that TgIST export is a process that occurs beyond the early stages of host cell infection. These findings have important implications regarding how this highly successful parasite maintains persistent infection of its host.

## Abstract (precis)

*Toxoplasma* bradyzoites persist within tissue cysts and are refractory to current treatments, serving as a reservoir for acute complications in settings of compromised immunity. Much remains to be understood regarding how this life stage successfully establishes and maintains persistent infection. In this study, we investigated whether the export of parasite effector proteins into the host cell occurs during the development of *in vitro* tissue cysts. We quantified the presence of four previously described effectors in host cell nuclei at different time points after bradyzoite differentiation and found that they accumulated largely during the early stages of infection. Despite a decline in nuclear accumulation, we found that one of these effectors still mediated its function after prolonged infection with bradyzoites, and we provide evidence that this effector is exported beyond early infection stages. These findings suggest that effector export from within developing tissue cysts provides one potential mechanism by which this parasite achieves chronic infection.

## INTRODUCTION

The intracellular parasite Toxoplasma gondii is estimated to infect up to one-third of the global human population (1). The success of this pathogen can be partially attributed to its flexible life cycle, in which a wide variety of hosts can be infected and transmit the latent life stage following predation by another organism (2). During acute infection, the tachyzoite life stage replicates quickly and robustly, disseminating to various tissues of the body (3). Although the host immune response can typically clear the majority of tachyzoites and overcome acute infection, a subset of parasites differentiate into the slowly growing life stage termed the bradyzoite, which persists into chronic or latent infection (4). Bradyzoites persist within their host for an indefinite period, residing predominately in muscle tissue and the brain (4). The mechanisms by which bradyzoites manage to evade the host immune system and to manipulate their host cell to optimize survival remain unclear and understudied.

Within the host cell, bradyzoites reside in a specialized vacuole termed the tissue cyst. Tissue cysts are typified by an amorphous collection of proteins and glycoconjugates termed the cyst wall, which forms underneath the limiting membrane, or cyst membrane, of the tissue cyst (5). Proteomic studies of the cyst wall from tissue cysts induced *in vitro* identified Myc-regulating protein 1 (MYR1) as a putative cyst wall protein (6). MYR1 was shown previously to be involved in the process of parasite protein translocation from the parasitophorous vacuole into the host cell during tachyzoite infection (7). Thus far, exported effector proteins that have been identified to be secreted in this manner include GRA16 (8), GRA18 (9), GRA24 (10), TgIST (11, 12), and HCE1/TEEGR (13, 14). In the context of tachyzoite infection, these effectors have been shown to bind to host cell proteins, translocate into the host nucleus (GRA18 remains in the host cytoplasm), and affect host cell signaling pathways to favor the parasite (15). GRA28 is an additional protein identified by proximity-based biotinylation that has been shown to localize to the host cell nucleus under tachyzoite growth conditions (16), although the host cell targets of this protein have yet to be identified.

Given the presence of MYR1 protein in the cyst wall, we hypothesized that bradyzoites retain the capacity to continually export proteins from within tissue cysts, so as to maintain constant control of the host cell. We set about testing this hypothesis by first confirming the cyst wall localization of MYR1. After GRA16, GRA24, GRA28, and TgIST were epitope tagged at their endogenous loci, we found, by immunofluorescence assays (IFAs), that the intensity of these effectors in the host nucleus declined over time in human fibroblasts containing individual vacuoles with differentiating parasites. A similar pattern of export was observed in fibroblasts infected with parasites further along the bradyzoite differentiation continuum, as well as during infection of mouse primary cortical neurons. We found that renewed nuclear effector accumulation did not occur during a tachyzoite superinfection in host cells containing older tachyzoite or bradyzoite vacuoles, indicating that declining levels of ROP17 are likely not the cause of the observed pattern of effector export. Despite the observed decline of effector export, an inducible knockout (KO) approach revealed that host nuclear TgIST-3×hemagglutinin (3×HA) was not detectable during the later phases of infection when deleted 1 day postinfection (p.i.), indicating that TgIST export continues to occur beyond day 1 p.i.

## RESULTS

**FIG 1:**
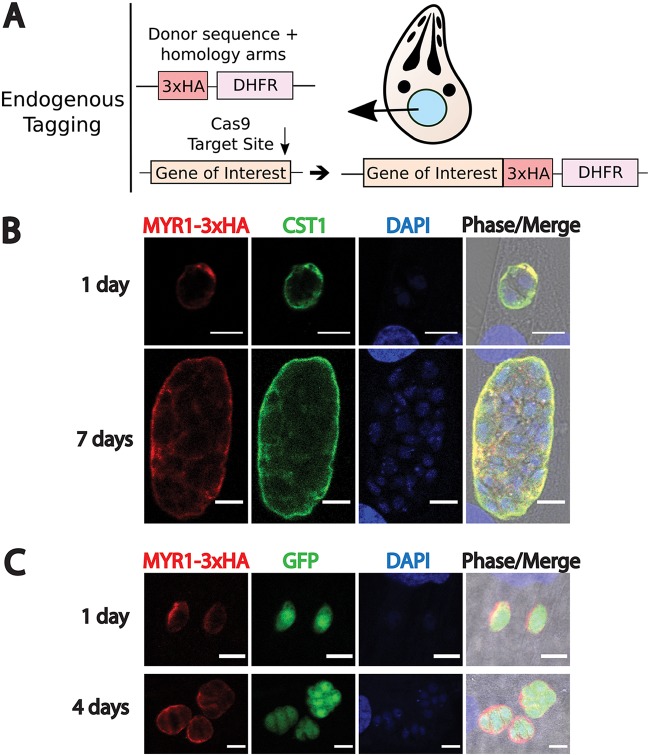
Endogenous tagging and IFA of MYR1-3×HA reveal that MYR1 is secreted into the vacuole during bradyzoite differentiation and after bradyzoite infection. (A) Diagram illustrating the Cas9 strategy to introduce a 3×HA epitope tag, the HXGPRT 3′ UTR, and a DHFR resistance cassette at the C terminus of the gene of interest. Note that the Cas9 target site was located in either the C terminus or 3′ UTR of a gene, and the donor sequence containing at least 30 bp of homology to endogenous sequences on both ends was amplified by PCR. See Table S1 for a list of the primers used. (B and C) Representative images of MYR1-3×HA localization (red) in vacuoles undergoing bradyzoite differentiation following tachyzoite invasion (B) or in vacuoles following invasion with parasites further along the bradyzoite differentiation path (C). Bradyzoite differentiation was determined by the SalmonE antibody to glycosylated CST1 (green in B), whereas anti-GFP antibody was used to identify differentiated bradyzoites expressing GFP under the control of the bradyzoite-specific LDH2 promoter (green in C). Nuclei were labeled with DAPI. Scale bar, 10 μm.

To validate the localization of MYR1 at the cyst wall of *in vitro* tissue cysts, the endogenous locus of MYR1 was epitope tagged at the C terminus with three copies of the HA tag (3×HA) in the PruΔ*ku80* strain, using CRISPR/Cas9 (Fig. 1A). After clonal populations of endogenously tagged parasites were obtained, IFAs were performed with infected human foreskin fibroblast (HFF) cultures. Egressed tachyzoites were used for initial experiments, with bradyzoite differentiation induced at the time of invasion by replacement of growth medium with low-serum alkaline medium prior to the addition of parasites, followed by subsequent culture in an ambient CO_2_ incubator. MYR1 was detected in the nascent cyst wall of vacuoles undergoing differentiation as early as 1 day p.i. and up to 7 days p.i., as determined by colocalization with glycosylated CST1 (Fig. 1B), an early marker of bradyzoite differentiation (17). MYR1 cyst wall localization was also evident at time points between 2 and 6 days p.i. (see Fig. S1 in the supplemental material). To determine whether cysts formed by parasites further along the bradyzoite differentiation path also contained MYR1, egressed parasites cultured *in vitro* under bradyzoite-inducing conditions for 6 days were used to infect a new HFF monolayer. Using green fluorescent protein (GFP) as a marker of bradyzoite differentiation (by virtue of GFP expression driven by the bradyzoite-specific LDH2 promoter in the PruΔ*ku80* strain [18]), MYR1 was shown to be expressed and secreted by these *in vitro*-derived bradyzoites upon infection of new host cells under bradyzoite-inducing conditions, at both 1 and 4 days p.i. (Fig. 1C).

10.1128/mBio.00046-20.2 FIG S1 Immunofluorescence images of MYR1-3×HA-tagged parasites during bradyzoite differentiation 2 to 6 days p.i. Representative images of MYR1-3×HA localization (red) in vacuoles undergoing bradyzoite differentiation, as determined with the SalmonE antibody to CST1 (green), are shown. Nuclei were labeled with DAPI. Scale bar, 10 μm. Download FIG S1, PDF file, 0.02 MB. Copyright © 2020 Mayoral et al. 2020 Mayoral et al. This content is distributed under the terms of the Creative Commons Attribution 4.0 International license.

10.1128/mBio.00046-20.1 TABLE S1 List of primers used in this study for CRISPR/Cas9 tagging of genes and for TgIST-GeneSwap-DiCre construction. Download Table S1, DOCX file, 0.02 MB. Copyright © 2020 Mayoral et al. 2020 Mayoral et al. This content is distributed under the terms of the Creative Commons Attribution 4.0 International license.

**FIG 2:**
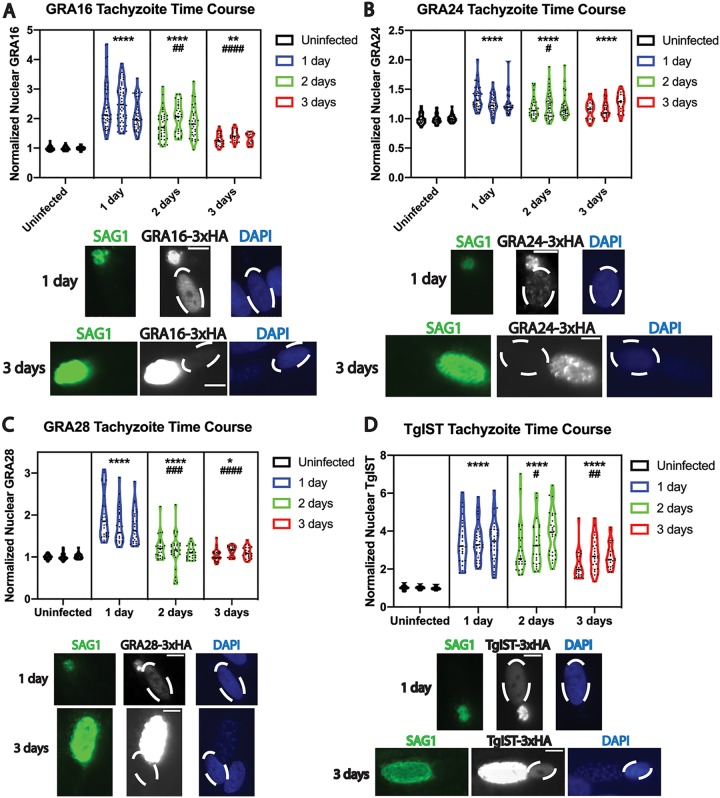
Quantitative analysis of exported effector fluorescence intensity in fibroblast host cell nuclei during tachyzoite infection reveals gradual declines in effector export over time. Violin plots and representative images of effector fluorescence intensity in infected fibroblast nuclei for GRA16-3×HA (A), GRA24-3×HA (B), GRA28-3×HA (C), and TgIST-3×HA (D), during the course of tachyzoite infection, are shown. Tachyzoite vacuoles were identified with antibody to SAG1 (green). Nuclei were labeled with DAPI (blue), and infected host cell nuclei are indicated by dashed ovals. Scale bar, 10 μm. The mean gray values measured from host nuclei infected with single vacuoles were obtained from at least 15 fields of view and were normalized to baseline fluorescence, measured from the nuclei of uninfected host cells. Three biological replicates are plotted for each time point, and mean gray values from at least 20 nuclei were measured for each replicate. Asterisks indicate a statistically significant increase (****, *P* < 0.0001; **, *P* < 0.01; *, *P* < 0.05), compared to the uninfected group, whereas hashtag symbols indicate a statistically significant decrease (####, *P* < 0.0001; ###, *P* < 0.001; ##, *P* < 0.01; #, *P* < 0.05) in infected groups, compared to 1 day p.i.

Applying the same Cas9 epitope-tagging strategy as used for MYR1 (Fig. 1A), the C termini of GRA16, GRA24, GRA28, and TgIST were epitope tagged separately in the PruΔ*ku80* strain with the 3×HA tag. To assess effector export in these strains first under tachyzoite growth conditions, IFAs with fibroblast monolayers infected with tachyzoites of each effector-tagged strain were performed after cultures were fixed in triplicate at 1, 2, and 3 days p.i. In these experiments, effector intensity in host nuclei was exclusively measured in host cells containing single tachyzoite vacuoles at each time point. As reported previously, each of these effectors was detectable in host nuclei and was found to be significantly above baseline fluorescence levels (uninfected fibroblast nuclei, normalized background fluorescence value of 1) at 1 day p.i. (Fig. 2A to D, asterisks). Although the nuclear intensities of all effectors were significantly above baseline up to 3 days p.i., the effectors demonstrated different degrees of significant decline in host nuclei at later time points, compared to their respective average values measured at 1 day p.i. (Fig. 2A to D, hashtag symbols), indicating that these effectors did not continuously accumulate in the host nucleus during the course of tachyzoite infection.

**FIG 3:**
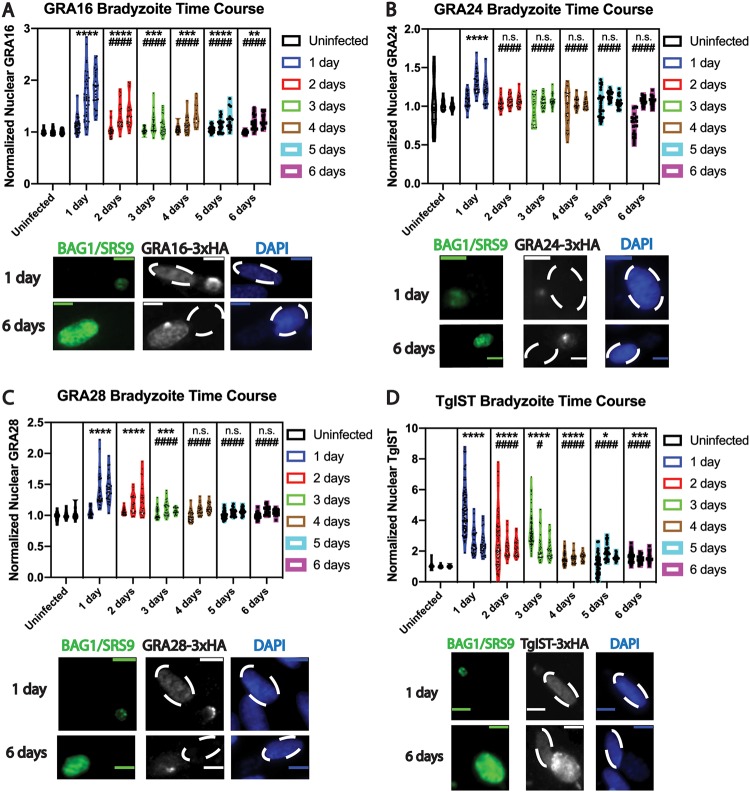
Quantitative analysis of exported effector fluorescence intensity in fibroblast host cell nuclei during bradyzoite differentiation reveals declines in effector export over time. Violin plots and representative images of effector fluorescence intensity in infected fibroblast nuclei for GRA16-3×HA (A), GRA24-3×HA (B), GRA28-3×HA (C), and TgIST-3×HA (D), during the course of bradyzoite infection, are shown. Bradyzoite differentiation was probed with antibody to BAG1 at 1 day p.i. or with antibody to SRS9 at 6 days p.i. (both green). Nuclei were labeled with DAPI (blue), and infected host cell nuclei are indicated by dashed ovals. Scale bar, 10 μm. Measurements were made from three replicate experiments at each time point, from infected nuclei containing a single differentiating vacuole at each time point, as described for Fig. 2. The same mean gray value normalization approach and symbol conventions to indicate significant increases from 1 day p.i. as in Fig. 2 were used. Asterisks indicate a statistically significant increase (****, *P* < 0.0001; ***, *P* < 0.001; **, *P* < 0.01; *, *P* < 0.05), compared to the uninfected group, whereas hashtag symbols indicate a statistically significant decrease (####, *P* < 0.0001; #, *P* < 0.05) in infected groups, compared to 1 day p.i.; n.s., nonsignificant difference, compared to uninfected baseline values.

After confirmation of previously described tachyzoite effector export patterns, IFAs with the four tagged effector strains were performed under bradyzoite growth conditions, starting with egressed tachyzoites and inducing bradyzoite differentiation at the time of infection, as described above. Monolayers were fixed daily from 1 day to 6 days p.i. The average fluorescence intensities of each effector were measured in the nuclei of host cells containing a single developing tissue cyst, ascertained by positive staining of either BAG1 (1 to 3 days p.i.) or SRS9 (4 to 6 days p.i.), both markers of bradyzoite differentiation (19, 20) (Fig. 3A to D). All effectors were detectable in host nuclei at the earliest time point (1 day p.i.) and were found to be significantly above average uninfected nuclear fluorescence intensity (i.e., the background uninfected normalized fluorescence value of 1) (Fig. 3A to D). Similar to measurements made with tachyzoite-infected cells, the average host nuclear intensities of each effector significantly declined at later time points, compared to intensities at 1 day p.i. (Fig. 3A to D). A divergence was noted, in which effectors persisted in host nuclei at relatively late time points after bradyzoite infection. Whereas TgIST and GRA16 levels remained significantly elevated, compared to baseline levels, in host nuclei up to 6 days p.i. (Fig. 3A and D), GRA24 and GRA28 levels were not significantly elevated starting at 2 days and 4 days p.i., respectively (Fig. 3B and C). Despite their apparent absence in the host cell nucleus at later time points, GRA24 and GRA28 fluorescence was evident in differentiating bradyzoite vacuoles up to 6 days p.i. (Fig. 3B and C, representative images), indicating that a secreted pool of GRA24 and GRA28 remained detectable in the vacuole, despite not being exported to the host cell nucleus, during the course of bradyzoite differentiation in this infection model.

**FIG 4:**
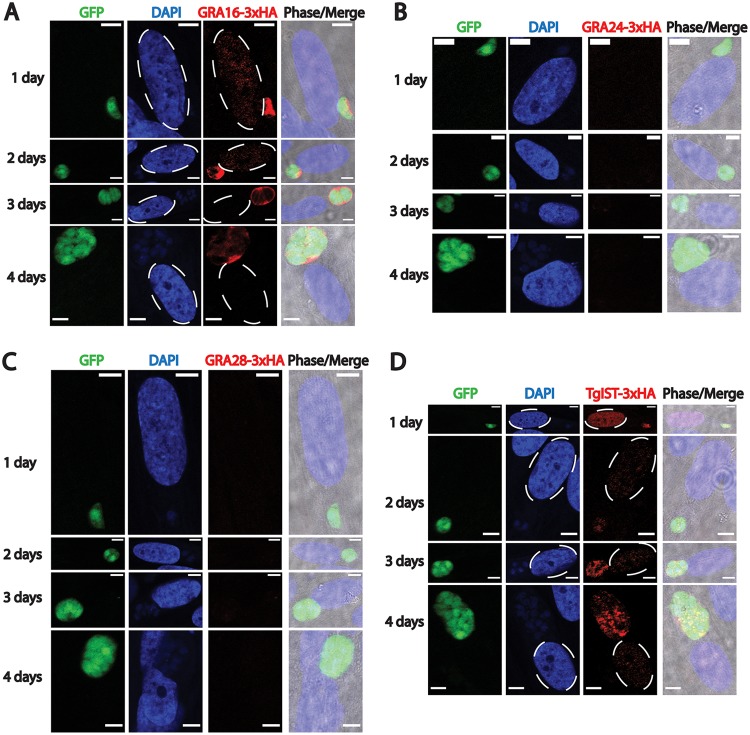
Immunofluorescence images of exported effector localization following infection with *in vitro*-derived bradyzoites reveal differential expression of certain effectors. Representative images of GRA16-3×HA (A), GRA24-3×HA (B), GRA28-3×HA (C), and TgIST (D) localization (all in red), after infection of fibroblasts with egressed bradyzoites (e.g., parasites further along the bradyzoite differentiation path obtained *in vitro*, as determined by anti-GFP antibody staining [green]), are shown. Nuclei were labeled with DAPI (blue), and infected host cell nuclei are indicated by dashed ovals. Images were captured from fibroblasts infected with a single vacuole. GRA16 and TgIST are detected in infected host cell nuclei at 1 and 2 days p.i., with TgIST being readily detectable in host cell nuclei up to 4 days p.i. GRA24 and GRA28 expression was undetectable. Scale bar, 10 μm.

Quantitation of effector intensity in fibroblast nuclei at various time points demonstrated a decline as a function of time during differentiation of tachyzoites to bradyzoites. We were interested in determining whether a similar pattern was observed when infection was initiated by egressed bradyzoites harvested from *in vitro* bradyzoite-induced parasite cultures (as described above for MYR1 experiments). IFAs with HFFs infected with egressed *in vitro*-induced bradyzoites revealed similar patterns of effector localization with respect to GRA16 and TgIST. GRA16 was detectable in the host nucleus at 1 and 2 days p.i. and appeared to decline in intensity at later time points (Fig. 4A), whereas TgIST was readily detectable at all time points (up to 4 days p.i.) (Fig. 4D). Intriguingly, GRA24 and GRA28 were not expressed at any time points using this infection model (Fig. 4B and C). The lack of expression of these two proteins in parasites further along the bradyzoite differentiation path is supported by transcriptomic data deposited in ToxoDB (www.toxodb.org), in which several bradyzoite data sets demonstrate few to no transcripts for these two genes, suggesting that small amounts of these proteins, if any, are translated in this life stage. Hence, this finding demonstrates that the *in vitro* model of bradyzoite differentiation used here reflects data obtained from *in vivo* data sets.

**FIG 5:**
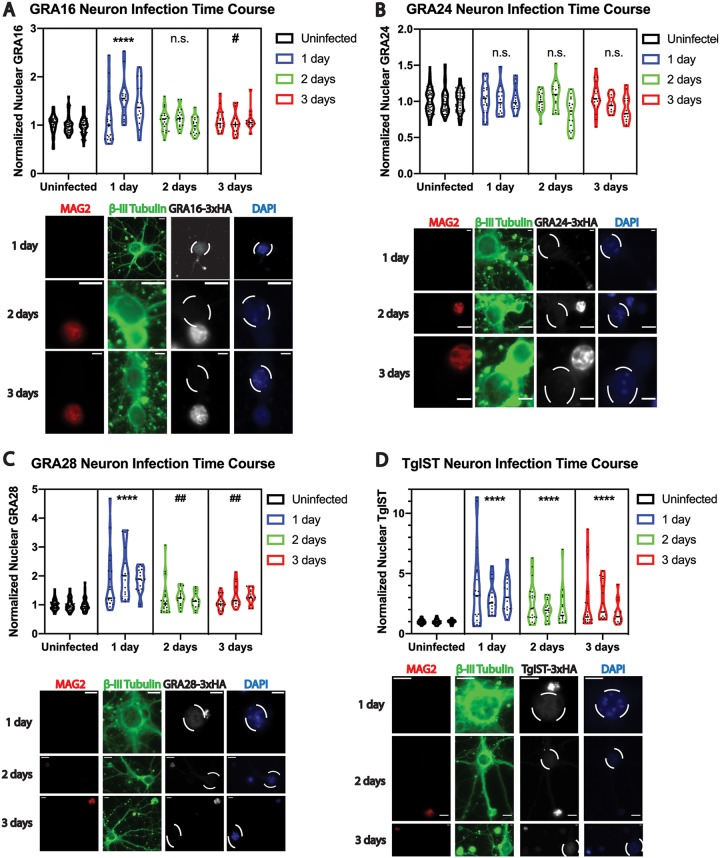
Quantitative analysis of exported effector fluorescence intensity in mouse primary cortical neuron nuclei reveals similar patterns of export, compared to fibroblast infection. Violin plots and representative images of effector fluorescence intensity in infected primary neuron nuclei for GRA16-3×HA (A), GRA24-3×HA (B), GRA28-3×HA (C), and TgIST-3×HA (D), during the course of infection, are shown. Bradyzoite differentiation was determined with antibody to MAG2 (TGME49_209755) at 1, 2, and 3 days p.i., although MAG2-positive vacuoles were not observed at 1 day p.i. Neurons were identified by positive β-III tubulin staining (green). Nuclei were labeled with DAPI (blue), and infected host cell nuclei are indicated by dashed ovals. Scale bar, 10 μm. Measurements were made from three replicate experiments at each time point, from infected nuclei containing a single vacuole at each time point. Mean gray values were normalized to uninfected nuclei, and symbol conventions to indicate significant increases from uninfected values or significant decreases from 1 day p.i. as in Fig. 2 were used. Asterisks indicate a statistically significant increase (****, *P* < 0.0001), compared to the uninfected group, whereas hashtag symbols indicate a statistically significant decrease (##, *P* < 0.01; #, *P* < 0.05) in infected groups, compared to 1 day p.i.; n.s., nonsignificant difference, compared to uninfected baseline values.

We were interested in measuring effector intensities in the nuclei of primary neurons, because neurons most frequently harbor tissue cysts in the mouse brain during chronic infection (21). Cortical neurons were harvested from mouse embryos after 14 days of gestation (embryonic day 14 [E14]) and were cultured *in vitro* on poly-l-lysine-coated coverslips for 14 days prior to infection. Egressed tachyzoites from each effector-tagged strain were then used to infect separate neuron cultures, which were fixed for IFAs at 1, 2, and 3 days p.i. Staining with a monoclonal antibody to a bradyzoite-secreted protein that we have named MAG2 (gene identification no. TGME49_209755) (34) confirmed the occurrence of bradyzoite differentiation at 2 and 3 days p.i. (Fig. 5A to D). The nuclear effector intensities at 2 and 3 days p.i. were determined in neurons infected with a single MAG2-positive vacuole, while nuclear intensities quantified at 1 day p.i. were obtained from infected neurons with single vacuoles, as MAG2 expression was not observed at 1 day p.i. in neurons. Using β-III tubulin as a neuron-specific marker, we found that host nuclear GRA24 was largely undetectable above baseline uninfected nuclear fluorescence at all time points, despite vacuolar expression (Fig. 5B). Of note, GRA24 export into neuron nuclei was detectable when the host neuron contained more than one vacuole at 1 day p.i. (data not shown). Similar to findings observed in HFF host cells, GRA16 and GRA28 export in neurons was significantly elevated above baseline at 1 day p.i. and declined to baseline levels thereafter (Fig. 5A and C). TgIST levels remained significantly above baseline in host neuron nuclei at all time points inspected, displaying a trend of declining host nuclear intensity at 2 and 3 days p.i. (Fig. 5D). Thus, parasites retained the capacity to export each of these four effectors into the host nucleus during neuron infection, exhibiting a pattern of export similar to that observed in HFF host cells.

**FIG 6:**
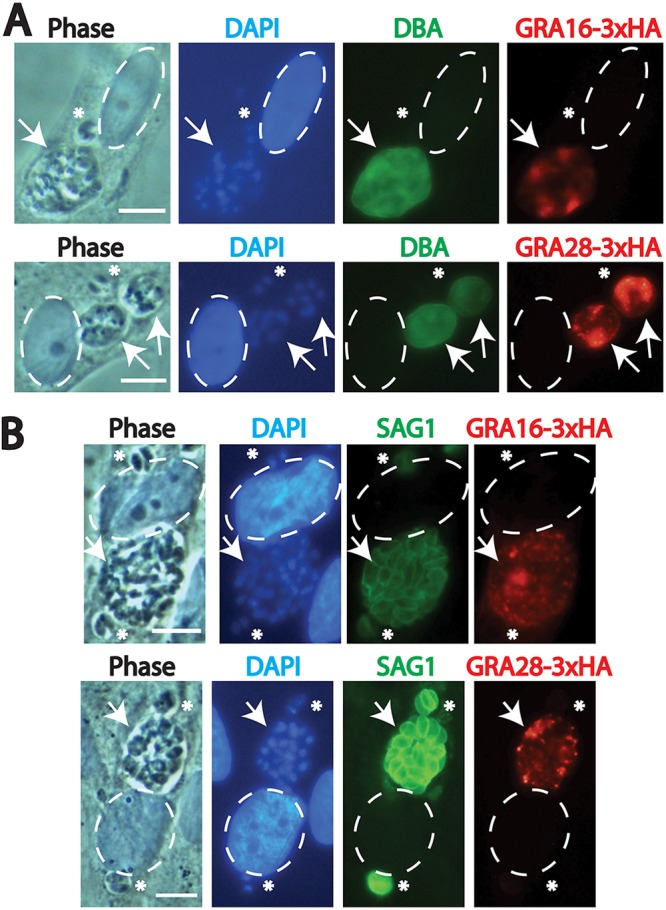
Immunofluorescence images of GRA16 and GRA28 localization following superinfection with non-epitope-tagged T. gondii tachyzoites demonstrate no renewed effector export. (A) Representative images of GRA16-3×HA and GRA28-3×HA localization (both in red) 1 day after tachyzoite invasion and 8 days after differentiation (tachyzoites were introduced 7 days after differentiation). Differentiated vacuoles are indicated by DBA-FITC staining (green). Nuclei were labeled with DAPI (blue), and infected host cell nuclei are indicated by dashed ovals. Arrows indicate the bradyzoite-containing vacuoles with vacuolar GRA16 or GRA28, whereas asterisks indicate tachyzoite vacuoles in the same host cell. Neither GRA16 nor GRA28 is exported from differentiated vacuoles into the host cell nucleus 1 day after tachyzoite invasion. Scale bar, 10 μm. (B) Representative images of GRA16-3×HA and GRA28-3×HA localization (both in red) 3 days after the initial infection and 1 day after tachyzoite superinfection (tachyzoites were introduced 2 days p.i.). Older vacuoles (arrows) from the initial infection are larger than vacuoles from the superinfection (asterisks), although both young and older tachyzoite vacuoles are labeled by SAG1 (green). Nuclei were labeled with DAPI (blue), and infected host cell nuclei are indicated by dashed ovals. Scale bar, 10 μm.

The gradual decline of each exported effector observed during both tachyzoite infection and bradyzoite infection suggested that some factor(s) affecting effector export decreased in quantity or function during the course of vacuolar development. We sought to determine the mechanism behind this observation, reasoning that it could be due to a feature shared by older bradyzoite and tachyzoite vacuoles. Recent work demonstrated that catalytically active ROP17 is required for the translocation of protein effectors across the parasitophorous vacuole membrane (PVM) (22). ROP17, along with other rhoptry-derived proteins, is secreted at the time of host cell invasion and ultimately localizes to the cytosolic face of the PVM. We speculated that declining levels of PVM-associated ROP17 during the course of intracellular infection may be the cause of declining effector translocation at later time points. To test this, we superinfected HFF monolayers containing parasites that had been cultured under bradyzoite differentiation conditions for 7 days with a batch of egressed PruΔ*ku80* strain tachyzoites that did not express the 3×HA epitope tag. We allowed this superinfection to progress for 1 more day under bradyzoite differentiation conditions and then performed IFAs with the monolayers, looking for a burst of host nuclear GRA16 or GRA28 in cells containing one differentiated vacuole (determined by Dolichos biflorus agglutinin [DBA]-fluorescein isothiocyanate [FITC] staining) and one new vacuole from superinfection. We found that no renewed GRA16 or GRA28 translocation was apparent under these conditions (Fig. 6A) even when HFFs contained multiple new vacuoles and a single bradyzoite vacuole, suggesting that the delivery of ROP17 from a new invasion event does not allow for the renewed export of vacuolar GRA16 and GRA28 from older vacuoles. The same results were obtained from an identical experiment performed under tachyzoite growth conditions, in which tachyzoite-infected host cells were superinfected at 2 days p.i. and fixed at 3 days p.i. (Fig. 6B). Other groups demonstrated previously that ROP17 provided by such superinfection reliably restored the activity of ROP17 on the parasitophorous vacuole (22; J. Boothroyd, personal communication). Thus, there are likely different mechanisms, either intravacuolar or extravacuolar, that restrict translocation in older vacuoles.

**FIG 7:**
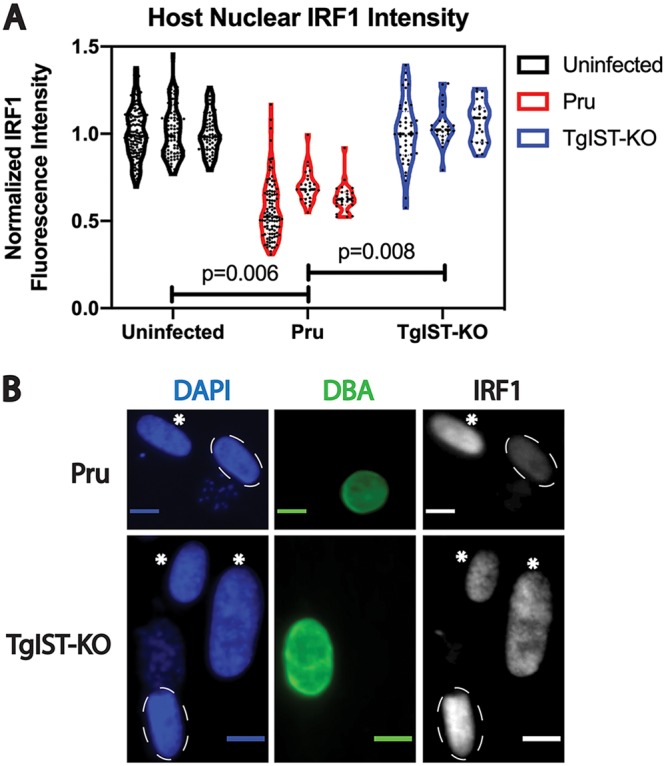
Quantitation and immunofluorescence images of IRF1 upregulation in IFN-γ-stimulated fibroblasts infected with either Pru or TgIST-KO demonstrate that IRF1 suppression is TgIST dependent during bradyzoite infection. (A) Violin plots of IRF1 intensity in the nuclei of uninfected fibroblasts or infected fibroblasts containing either PruΔ*ku80* or Pru TgIST-KO parasites, measured from three replicates. The mean fluorescence intensity of IRF1 was normalized to the average values obtained from uninfected nuclei. The IRF1 intensity values plotted for the uninfected group were obtained from both PruΔ*ku80*- and Pru TgIST-KO-infected cultures for each replicate. (B) Representative images of fibroblasts infected initially with egressed tachyzoites from the PruΔ*ku80* strain or the Pru TgIST-KO strain, 7 days p.i. under bradyzoite-inducing conditions. Fibroblasts were exposed to IFN-γ at a concentration of 100 U/ml for 6 h at 7 days p.i. IFN-γ signaling was probed with anti-IRF1 antibody (red), and bradyzoite differentiation was determined by staining for DBA-FITC (green), a cyst wall marker. Nuclei were labeled with DAPI (blue), and images were captured from fibroblasts infected with a single vacuole. Notable IRF1 expression is observed in uninfected nuclei (asterisks), whereas IRF1 expression is attenuated in infected host cell nuclei (dashed ovals) containing Pru parasites but not TgIST-KO parasites. Scale bar, 10 μm.

Robust TgIST export is notable both during tachyzoite infection (Fig. 2D) and during the bradyzoite infection models used in this study (Fig. 3D and 4D). TgIST has been shown to suppress the host cell response to interferon gamma (IFN-γ), preventing the upregulation of genes downstream of this signaling pathway, such as interferon response factor 1 (IRF1). Given the known function of TgIST and the findings obtained by IFA, we hypothesized that cells infected with differentiating bradyzoites remained unresponsive to exogenous IFN-γ at relatively late time points p.i. Using IRF1 as a probe for IFN-γ signaling, we measured host nuclear IRF1 fluorescence intensity in HFFs harboring a single tissue cyst and stimulated with recombinant IFN-γ at 7 days p.i. The results from multiple IFAs demonstrated that significant attenuation of IRF1 upregulation occurred in HFFs infected with parasites of the PruΔ*ku80* strain, compared to uninfected cells and cells infected with TgIST-KO parasites (Fig. 7A and B). Hence, in the context of prolonged bradyzoite infection, TgIST appears to be at least partially responsible for suppressing IFN-γ signaling in the host cell.

**FIG 8:**
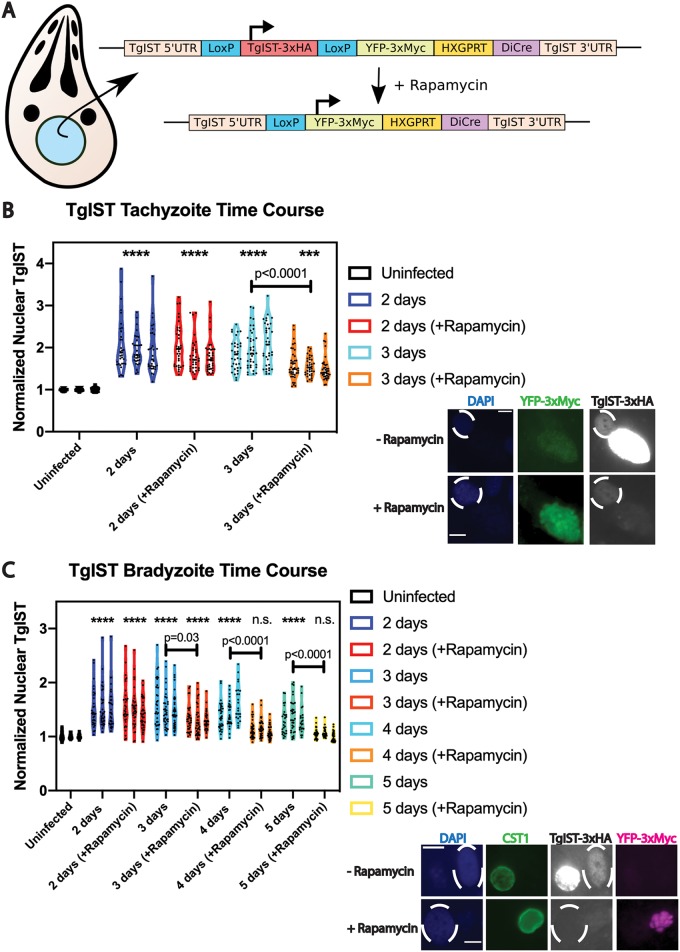
TgIST-GeneSwap-DiCre parasites allow for conditional KO of ectopic TgIST-3×HA, revealing that the stability of TgIST in host fibroblast nuclei is limited to several days during the course of bradyzoite differentiation. (A) Illustration of the inducible TgIST-3×HA KO strategy. Upon the addition of rapamycin, the TgIST-3×HA transgene is deleted, allowing for the expression of YFP-3×Myc driven by the TgIST promoter and 5′ UTR sequence. Both constitutively expressed DiCre fragments and an HXGPRT-selectable marker are included in the same construct. (B) Violin plots of normalized TgIST fluorescence intensity in host fibroblast nuclei during the course of tachyzoite infection. Measurements were made from three replicate experiments at each time point, from infected nuclei containing a single vacuole at each time point. Mean gray values were normalized to uninfected nuclei. Symbol conventions to indicate significant increases from uninfected values (asterisks) as in Fig. 2 were used. A significant decrease in normalized TgIST intensity was observed in rapamycin-treated coverslips, compared to untreated coverslips, at 3 days p.i. Representative images of TgIST-GeneSwap-DiCre parasites at 3 days p.i., with or without a 24-h 50 nM rapamycin pulse exposure 1 day p.i., allowing for an initial TgIST secretion event, are shown. Nuclei were labeled with DAPI (blue), YFP-3×Myc reporter with Myc tag antibody (green), and TgIST-3×HA with HA antibody. Infected host cell nuclei are indicated by dashed ovals. Scale bar, 10 μm. (C) Violin plots of normalized TgIST fluorescence intensity in host fibroblast nuclei during the course of bradyzoite infection. Measurements were made from three replicate experiments at each time point, from infected nuclei containing a single vacuole at each time point. Mean gray values were normalized to uninfected nuclei. Symbol conventions to indicate significant increases from uninfected values (asterisks) as in Fig. 2 were used. A significant decrease in normalized TgIST intensity was observed in rapamycin-treated coverslips, compared to untreated coverslips, at 3, 4, and 5 days p.i. Host nuclear TgIST levels were not found to be significantly elevated above uninfected values in rapamycin-treated coverslips at 4 and 5 days p.i. (n.s.). Representative images of TgIST-GeneSwap-DiCre parasites 5 days after bradyzoite differentiation, with or without a 24-h 50 nM rapamycin pulse exposure 1 day p.i., are shown. Bradyzoite differentiation was assessed with the SalmonE antibody to glycosylated CST1 (green), nuclei were labeled with DAPI (blue), TgIST-3×HA was labeled with HA antibody, and the YFP-3×Myc reporter was labeled with Myc tag antibody (magenta). Infected host cell nuclei are indicated by dashed ovals. Scale bar, 10 μm.

The notable persistence of TgIST in the host nucleus at 6 days p.i., and the prolonged effects mediated by this protein in the host cell, raised the possibility that TgIST could be an effector that is continuously exported, albeit at reduced efficiency during late infection. Alternatively, TgIST persistence at late time points could be due to a relatively large bolus of TgIST export during the early phase of infection. To address these possibilities, we sought to determine the stability of TgIST in the host nucleus by using a previously described inducible KO strategy, in which genetic deletion is achieved by using exogenous rapamycin to dimerize Cre fragments and to induce Cre enzymatic activity (23). A single plasmid containing the following was constructed after several cloning steps: floxed TgIST-3×HA-tagged locus driven by the endogenous TgIST promoter and TgIST 5′ untranslated region (UTR), yellow fluorescent protein (YFP)-3×Myc-tagged reporter protein (the expression of which is induced only after Cre-mediated removal of the TgIST coding sequence), and open reading frames (ORFs) encoding two “DiCre” fragments (Cre59 and Cre60), among other elements (Fig. 8A). PruΔ*ku80* strain tachyzoites were transfected with the aforementioned linearized construct (TgIST-GeneSwap-DiCre). PCR analysis of genomic DNA from subcloned parasites revealed that the construct had inserted as a second copy into the parasite genome (data not shown); therefore, in subsequent experiments with the TgIST-GeneSwap-DiCre parasites, the ectopic 3×HA-epitope-tagged copy of TgIST driven by its own endogenous promoter was detected by IFA and then deleted by DiCre. To evaluate the DiCre parasites, cultures infected for 1 day under bradyzoite growth conditions were exposed to 50 nM rapamycin for 24 h, after which rapamycin-containing medium was removed and replaced with fresh medium. IFAs of these cultures at 5 days p.i. revealed that the vast majority of TgIST-GeneSwap-DiCre parasites expressed the YFP-3×Myc reporter protein and only trace amounts of TgIST-3×HA, whereas, in the absence of rapamycin exposure, most TgIST-GeneSwap-DiCre parasites demonstrated robust TgIST-3×HA expression and expressed no YFP-3×Myc reporter protein (Fig. 8C, representative images).

A time course was determined for ectopic TgIST-3×HA expression in the TgIST-GeneSwap-DiCre parasites in both tachyzoites and bradyzoites. TgIST-GeneSwap-DiCre parasites were allowed to infect HFF monolayers under tachyzoite or bradyzoite growth conditions, and a rapamycin pulse was provided at 1 day p.i. IFAs were performed daily following the rapamycin pulse, to determine how host nuclear TgIST intensity changed, comparing rapamycin-treated and untreated TgIST-GeneSwap-DiCre parasites. In these experiments, only host cells containing YFP-3×Myc-positive parasite cells were analyzed in the rapamycin-treated group, whereas only host cells containing YFP-3×Myc-negative parasites were analyzed in the untreated group. Under tachyzoite growth conditions, a significant decrease in normalized nuclear TgIST levels was evident in host cells from rapamycin-treated coverslips, compared to untreated cultures, at 3 days p.i. (Fig. 8B): at all time points, however, TgIST was detectable above baseline fluorescence even after TgIST deletion at 1 day p.i. (Fig. 8B, asterisks), indicating at least some portion of TgIST protein exported during the first day of infection persisted in the host nucleus after 3 days of tachyzoite infection. Intriguingly, we found a similar decrease in TgIST nuclear intensity during the bradyzoite time course experiment at 3 days p.i., although host nuclear TgIST was no longer detectable above baseline fluorescence at 4 and 5 days p.i. (Fig. 8C). Rapamycin pulse exposure itself did not affect TgIST-3×HA stability in host nuclei, as experiments with endogenously TgIST-3×HA-tagged parasites without the DiCre transgene demonstrated TgIST host nuclear persistence above baseline at 3, 4, and 5 days p.i. with exposure to a 24-h rapamycin pulse at 1 day p.i. (Fig. S2). Hence, genetic deletion of ectopic TgIST on day 1 p.i. indicates that this protein is exported beyond day 1 and that, following export on day 1, TgIST can persist at some level in the host nucleus up to about 4 days p.i.

10.1128/mBio.00046-20.3 FIG S2 Rapamycin pulse exposure does not alter the observed stability of TgIST-3×HA in host fibroblast nuclei. (A) Violin plots of normalized TgIST fluorescence intensity in host fibroblast nuclei during the course of bradyzoite infection with the endogenously tagged PruΔ*ku80* TgIST-3×HA strain (constructed as in Fig. 1A). Host nuclear TgIST levels remained significantly elevated above uninfected values at 3, 4, and 5 days p.i. after a 24-h 50 nM rapamycin pulse exposure 1 day p.i. Measurements were made from three replicate experiments at each time point, from infected nuclei containing a single vacuole at each time point. Mean gray values were normalized to uninfected nuclei. Symbol conventions to indicate significant increases from uninfected values as in Fig. 2 were used. Asterisks indicate a statistically significant increase (****, *P* < 0.0001; ***, *P* < 0.001; **, *P* < 0.01; *, *P* < 0.05), compared to the uninfected group, whereas hashtag symbols indicate a statistically significant decrease (####, *P* < 0.0001; ###, *P* < 0.001; ##, *P* < 0.01; #, *P* < 0.05) in infected groups, compared to 1 day p.i. (B) Representative images of bradyzoite differentiation and nuclear TgIST intensity at 3, 4, and 5 days p.i. Bradyzoite differentiation was assessed with the SalmonE antibody to glycosylated CST1 (green), nuclei were labeled with DAPI (blue), and TgIST-3×HA was labeled with HA antibody. Infected host cell nuclei are indicated by dashed ovals. Scale bar, 10 μm. Download FIG S2, PDF file, 0.03 MB. Copyright © 2020 Mayoral et al. 2020 Mayoral et al. This content is distributed under the terms of the Creative Commons Attribution 4.0 International license.

## DISCUSSION

Our findings demonstrate that parasite protein translocation across the nascent cyst membrane is a feature of bradyzoite infection in both fibroblasts and neurons. We set about to test our initial hypothesis of continuous bradyzoite effector export after our previous finding of MYR1 as a putative cyst wall protein (6). The expression of MYR1 within *in vitro* tissue cysts at various time points was validated, as this protein was secreted into differentiating vacuoles regardless of whether the invading parasite more closely resembled a tachyzoite or a bradyzoite (Fig. 1; also see Fig. S1 in the supplemental material). The presence of MYR1 in differentiating tissue cysts clearly allows effector export to occur at the early stages of infection, although seemingly not at later stages of infection in the cases of GRA24 and GRA28 (Fig. 3B and C). The differences in host nuclear intensities between GRA16/TgIST and GRA24/GRA28 at late time points may reflect decreased transcription of GRA24 and GRA28 during bradyzoite differentiation, as documented by various bradyzoite data sets on ToxoDB. Intriguingly, we found that invasion by *in vitro*-derived egressed bradyzoites did not result in the export of either GRA24 or GRA28, as neither protein was expressed in this infection model (Fig. 4). This finding suggests that, depending on where the parasite lies on the bradyzoite differentiation continuum, a different arsenal of exported effectors may be utilized by the bradyzoite. This could allow the parasite to refine the manner of host cell manipulation. For example, a previous report linked GRA24 as a negative regulator of bradyzoite differentiation (24). In that context, GRA24 export from a differentiating vacuole could interfere with the process of bradyzoite and cyst maturation.

The export of all four effectors studied here could be detected in mouse primary cortical neurons, although GRA24 export was undetectable when analyses were limited to single-vacuole infections (Fig. 5B). This finding suggests that effector export may occur in this cell type during the establishment of chronic infections *in vivo*, although clearly further investigation is needed to determine the nature of parasite protein export from the parasitophorous vacuole *in vivo* during acute and chronic infections in sites such as skeletal muscle and brain. We found that, while similar patterns of export were identified in neurons, compared to fibroblast infection (robust export at 1 day p.i., followed by decline thereafter), the decline to baseline host nuclear fluorescence levels was more rapid in neurons for both GRA16 and GRA28. Because each of these effectors was detectable in the parasitophorous vacuole during neuron infection, we reason that this finding could reflect cell type-specific differences in nuclear effector stability or that effector export is less efficient in neurons. These findings may partially explain the previously reported cell type-dependent transcriptional responses to T. gondii in fibroblasts, neurons, astrocytes, and skeletal muscle cells (25).

It is apparent that all effectors accumulate within developing tissue cysts *in vitro* during the tachyzoite-to-bradyzoite transition, possibly due to decreased capacity or efficiency to export these proteins. This finding could reflect the maturation of the cyst wall and the function of the cyst wall as a barrier to large molecule transport, as observed previously using fluorescent dyes of various sizes (26). However, we do not exclude the possibility that these effectors may perform additional functions within the tissue cyst. The mechanism underlying the decline in effector export at later time points is unclear and may not occur uniquely in bradyzoite vacuoles, as the time course of tachyzoite infection revealed a similar pattern (Fig. 2). None of these protein effectors appeared to be differentially processed at late time points after bradyzoite differentiation when protein migration was assessed by immunoblotting (data not shown).

In light of the recently described role of the rhoptry protein ROP17 in the export of MYR-dependent effectors (22), we tested whether new infections with egressed tachyzoites (providing fresh ROP17 in *trans*) could initiate the translocation of effectors present in older tachyzoite or bradyzoite vacuoles, and no renewed export was detected (Fig. 6). Hence, declining ROP17 levels during infection do not appear to be the cause of export decline. In any case, the pattern of protein export observed for the effectors in this study suggests a common need shared by tachyzoites and bradyzoites in rewiring the host cell during the early stages of infection, inducing changes that may persist long after the degradation of these effectors in the host cell.

Despite an apparent decline in effector export, we were interested in determining how long TgIST persisted in fibroblast nuclei and, by extension, whether TgIST might be a continuously exported effector. We engineered a parasite strain expressing a floxed ectopic epitope-tagged copy of TgIST that could be deleted by inducible Cre excision, and we found that, when this copy was deleted at 1 day p.i., TgIST was no longer detectable during bradyzoite differentiation at 4 and 5 days p.i. (Fig. 8C). We reason that, if a bolus of TgIST exported during the first day of infection persists in host fibroblast nuclei for only 4 days and TgIST export above baseline typically can be detected up to 7 days p.i., then there are export events occurring at least 2 or 3 days p.i. Further experiments are needed to model TgIST export during bradyzoite infection in more detail. If TgIST export occurs at a basal level at late time points, this may position TgIST as a unique exported effector among the four studied here, as a previous study demonstrated the absence of GRA16 and GRA24 export into the host cell when expression was initiated 5 days after bradyzoite differentiation, using a Tet operator approach (27).

The prolonged suppression of IFN-γ signaling in bradyzoite-infected fibroblasts is not surprising, given the persistence of TgIST, compared to other effectors, in the host cell nucleus during bradyzoite infection. TgIST has been shown to simultaneously bind STAT1 and components of the Mi-2-NuRD protein complex, leading to altered chromatin marks at genomic loci recognized by STAT1 and preventing STAT1-mediated transcription (11, 12). TgIST-dependent attenuation of IFN-γ signaling was demonstrated in bradyzoite-infected fibroblasts (Fig. 7), suggesting that, despite declining levels of TgIST at later time points after infection, the altered chromatin state at STAT1-binding loci may be responsible for long-lasting STAT1 transcriptional repression in the infected host cell. As no robust increase of nuclear IRF1 levels was detected in neurons following IFN-γ exposure for various periods (data not shown), similar attenuation of IRF1 levels due to TgIST could not be demonstrated in primary cortical neurons. IFN-γ signaling in neurons is currently not well characterized, although studies with primary mouse hippocampal neuron cultures demonstrated an intact, albeit delayed, IFN-γ signaling pathway (28, 29). Indeed, this delayed neuronal signaling cascade downstream to IFN-γ may make this cell type more vulnerable to successful parasite infection and could partially explain why tissue cysts are most frequently found in neurons in the brain.

GRA16 export occurs early following *in vitro*-derived bradyzoite invasion of a new host cell. GRA16 has been shown to bind to herpesvirus-associated ubiquitin-specific protease (HAUSP) and PP2A in various human and mouse cell lines, and substantial evidence has been provided to show that GRA16 is likely involved in the G_2_/M-phase arrest of the infected host cell (8). It is known that, during chronic infections *in vivo*, bradyzoite cysts typically reside in neurons in the brain and in skeletal muscle cells in muscle tissue (21, 30), both cell types that are terminally differentiated with respect to their cell cycle. We speculate that GRA16 may play a more relevant role in bradyzoite infection of other cell types (e.g., intestinal epithelial cells) following carnivory of tissue containing tissue cysts, with host cell cycle control likely being more important for successful replication and dissemination in a new host. However, the possibility that GRA16 serves additional functions in terminally differentiated cell types, or potentially interacts with other host proteins in different cell types, cannot be excluded. In summary, the data presented here extend our knowledge of an intriguing method used by this parasite to manipulate the cells it infects, pointing toward one mechanism being responsible for the persistence of this organism during chronic infection of its host.

## MATERIALS AND METHODS

### Cell culture.

PruΔ*ku80*Δ*hxgprt* LDH2-GFP parasites (18) were continuously passaged in HFF (ATCC CRL-1634; Hs27) host cells, as described previously (31). TgIST-KO parasites were obtained as a kind gift from the laboratory of David Sibley (Washington University School of Medicine, St. Louis, MO). For all experiments using fibroblasts, bradyzoite induction was performed at the time of invasion, by replacing growth medium with bradyzoite induction medium (50 mM HEPES [pH 8.2] in Dulbecco’s modified Eagle’s medium [DMEM] supplemented with 1% fetal bovine serum, penicillin, and streptomycin) prior to infection with egressed tachyzoites at a multiplicity of infection of 1 for infection of confluent HFF monolayers on glass coverslips or a multiplicity of infection of 2 in T25 flasks. Bradyzoite induction cultures were incubated in a humidified 37°C incubator without CO_2_. For infection of coverslips, induction medium was changed every 2 days. To obtain egressed bradyzoites from T25 flasks, induction medium was changed only on the second day of infection. Parasites, i.e., egressed bradyzoites, were harvested from cultures 6 days postinduction, when over 95% of T. gondii demonstrated GFP expression in the culture.

Mouse primary cortical neurons were harvested from E14 mouse embryos obtained from pregnant C57BL/6 mice (ordered from Charles River). Dissections of E14 cortical neurons were performed as described previously (32). Following dissection, 30,400 cortical neurons were added to poly-l-lysine-coated glass coverslips on 24-well plates and later were cultured in neurobasal medium (Thermo Fisher) supplemented with GlutaMAX supplement (Thermo Fisher) and B-27 supplement (Gibco). After 4 days *in vitro*, cytarabine (ara-C) was added to each culture at a final concentration of 0.2 μM, to minimize contamination from dividing, nonneuronal cells. Cultures were maintained for up to 18 days by replacing one-half of the conditioned medium with fresh supplemented neurobasal medium every 7 days.

For TgIST-DiCre time course experiments, TgIST-GeneSwap-DiCre parasites (or control endogenously TgIST-3×HA-tagged parasites without the DiCre transgene) were allowed to infect HFF monolayers under tachyzoite growth conditions or were induced to differentiate into bradyzoites as described above. At 24 h p.i., tachyzoite or bradyzoite differentiation medium was replaced with fresh medium supplemented with 50 nM rapamycin. After 24 h of rapamycin exposure, cell medium was replaced with fresh medium without additional rapamycin, and infected cells were cultured in the absence of exogenous rapamycin thereafter.

### Cloning and parasite transfection.

To epitope tag the genes described in this study at their endogenous loci using CRISPR/Cas9, single guide RNAs targeting the C terminus or 3′ UTR of each gene of interest were cloned separately into the p-HXGPRT-Cas9-GFP plasmid backbone using kinase-ligase-DpnI (KLD) reactions, as described previously (33). Donor sequences for homology-mediated recombination were generated by amplifying a 3×HA tag, the 3′ UTR of hypoxanthine phosphoribosyltransferase (HXGPRT), and a dihydrofolate reductase (DHFR) minicassette to confer pyrimethamine resistance from the previously described pLIC-3HA-DHFR plasmid backbone (6) (Fig. 1A). Primers used to amplify the donor sequences also contained overhangs with at least 30 bp of homology to the C terminus or 3′ UTR of the gene of interest.

To engineer a floxed TgIST-3×HA vector that also contained a YFP-3×Myc reporter gene, a HXGPRT-selectable marker, and the Cre59 and Cre60 DiCre fragments under the control of robust eukaryotic promoters in a single vector (TgIST-GeneSwap-DiCre), several sequential Gibson assembly reactions were performed. Briefly, in the first assembly, a YFP ORF and a HXGPRT-selectable marker were PCR amplified from a GeneSwap plasmid backbone (a kind gift from Kami Kim) and concatenated using a forward primer containing a LoxP sequence and homology to the HXGPRT 3′ UTR sequence downstream of a pLIC-TgIST-3×HA construct containing the endogenous TgIST promoter and 5′ UTR and a reverse primer containing homology to a DiCre plasmid backbone containing the promoters, 3′ UTRs, and ORFs of the Cre59 and Cre60 DiCre fragments (a kind gift from Kami Kim). In the second assembly, the concatenated construct from the first assembly was PCR amplified and further concatenated to include a LoxP sequence upstream of the TgIST start codon, as well as an arm homologous to the endogenous 3′ UTR of TgIST downstream of the DiCre sequences. A KLD reaction was performed to introduce a 3×Myc epitope tag at the C terminus of the YFP ORF, using the second Gibson assembly reaction product. The final Gibson assembly was performed to reintroduce the Cre59 promoter, ORF, and 3′ UTR from the DiCre plasmid backbone after it was found to be absent in the second Gibson assembly product. A plasmid map of the final TgIST-GeneSwap-DiCre construct is available upon request. A full list of primers used for cloning and epitope tagging can be found in Table S1 in the supplemental material.

PruΔ*ku80Δhxgprt* tachyzoites were transfected with 7.5 μg Cas9 plasmid and 1.5 μg PCR-amplified donor sequence through electroporation in cytomix buffer. Transfected parasites were selected with 2 μM pyrimethamine for at least two passages, after which resistant parasites were subcloned by limiting dilution. For transfection of PruΔ*ku80Δhxgprt* tachyzoites with the TgIST-GeneSwap-DiCre vector, 7.5 μg Cas9 plasmid targeting the TgIST C terminus was cotransfected with 40 μg of the TgIST-GeneSwap-DiCre vector linearized with the ScaI restriction enzyme (NEB). Drug selection with DMEM containing 25 μg/ml mycophenolic acid and 50 μg/ml xanthine was performed 24 h posttransfection for the following 6 days before removal of selection medium and subcloning by limiting dilution after sufficient parasite egress was observed.

### Immunofluorescence assays.

Confluent HFF monolayers and primary neurons were cultured on glass coverslips and infected with either egressed tachyzoites or egressed bradyzoites differentiated *in vitro* following 6 days of growth under bradyzoite-inducing conditions. All infections occurred under bradyzoite-inducing conditions at the time of infection. For the IFN-γ stimulation experiments, HFF monolayers infected initially with tachyzoites and induced to differentiate into bradyzoites were stimulated with 100 U/ml IFN-γ at 7 days p.i. and fixed at 6 h poststimulation. All coverslips were fixed with 4% paraformaldehyde for 20 min at room temperature, permeabilized in a 0.2% Triton X-100-0.1% glycine solution for 20 min at room temperature, rinsed with phosphate-buffered saline, and blocked with 1% bovine serum albumin for either 1 h at room temperature or overnight at 4°C. Coverslips were next labeled as follows: HA-tagged proteins were detected with rat anti-HA monoclonal antibody 3F10 (1:200; Sigma), parasite cyst wall and cyst matrix with mouse SalmonE anti-CST1 antibody (1:500) and mouse anti-MAG2 antibody (1:500), respectively, bradyzoites with mouse anti-BAG1 (1:500) and rabbit anti-SRS9 (1:1,000), GFP with rabbit anti-GFP (1:500; Thermo Fisher), IRF1 with rabbit anti-IRF1 (Cell Signaling, 1:500), tachyzoite SAG1 with mouse anti-SAG1 (1:500; Thermo Fisher), and neurons with rabbit anti-β-III tubulin (1:1,000; a kind gift from David Sharp, Albert Einstein College of Medicine, New York, NY). Rabbit anti-Myc tag antibody (1:500; Cell Signaling Technologies) was used to detect YFP-3×Myc reporter expression in TgIST-DiCre-infected coverslips. Various combinations of Alexa 488-conjugated anti-rabbit IgG and anti-mouse IgG, Alexa 555- and Alexa 594-conjugated anti-rat IgG, and Alexa 633-conjugated anti-rabbit IgG antibodies were used as secondary antibodies, at a dilution of 1:1,000 (Thermo Fisher). 4′,6-Diamidino-2-phenylindole (DAPI) counterstain was used to label parasite and host cell nuclei (1:2,000). Coverslips were mounted in ProLong Gold anti-fade reagent (Thermo Fisher) and imaged using either a Leica SP8 confocal microscope, a Nikon Eclipse wide-field fluorescence microscope (Diaphot 300), or a Pannoramic 250 Flash III automated slide scanner (3DHistech).

### Quantitative image analysis.

For each replicate and time point and for every time course, at least 15 randomly selected fields of view either were obtained and exported from CaseViewer software (3DHistech) after images were acquired with a Pannoramic 250 automated slide scanner or were obtained from imaging with a Lecia SP8 confocal microscope. For each effector time course, identical exposure times were used to detect effector fluorescence intensity from each coverslip. Images obtained from either the confocal or slide scanner microscopes were viewed in ImageJ (NIH), and nuclei were segmented to generate regions of interest. Regions of interest (nuclei) were selected manually from fibroblasts or neurons that contained individual parasitophorous vacuoles, and the mean gray value was measured for each region of interest in the fluorescent channel used to detect each effector. Background fluorescence was determined by selecting nuclei from fibroblasts or neurons that did not contain parasitophorous vacuoles (uninfected) and measuring mean gray values as described above. To allow for comparisons between replicate experiments and across various time points, mean gray values measured from infected host nuclei were normalized by dividing each measured value by the average background fluorescence quantified from uninfected nuclei at each time point (i.e., from each coverslip). Normalized values were plotted using Prism 8 (GraphPad). Because each normalized mean gray value data set was shown not to exhibit a Gaussian distribution after testing for normality, the nonparametric Kruskal-Wallis test, with Dunn’s multiple-comparison test, was used to compare the means from three replicates for a given time point, to determine statistically significant differences between groups.
