# Supplementary figures and images for: In Vitro Characterization of Protein Effector Export in the Bradyzoite Stage of Toxoplasma gondii
Source: mBio. 2020 Mar 10;11(2):e00046-20. doi: 10.1128/mBio.00046-20 (PMC7064745; doi:10.1128/mBio.00046-20)

**MYR1-3xHA****CST1****DAPI****Phase/Merge****2 days**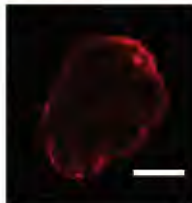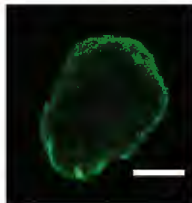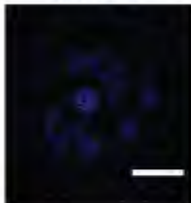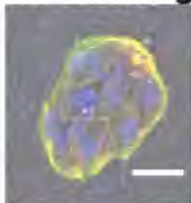**3 days**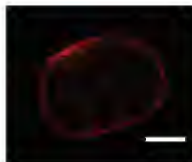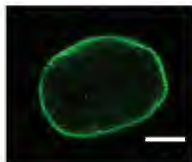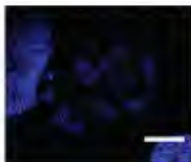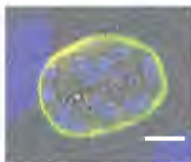**4 days**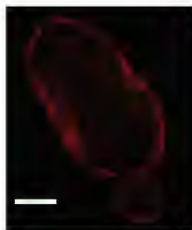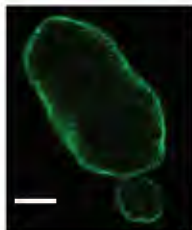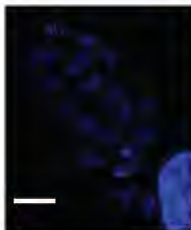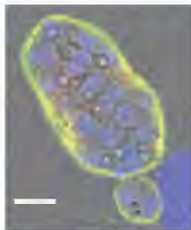**5 days**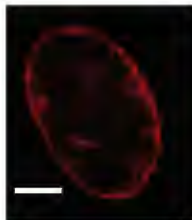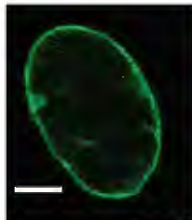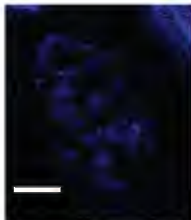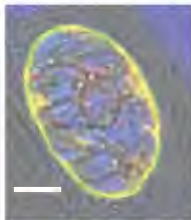**6 days**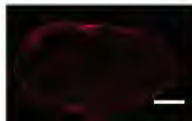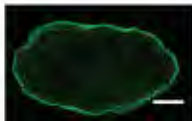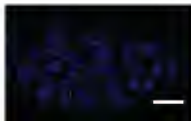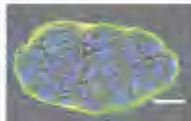

Supplement: FIG S1 [file mBio.00046-20-sf001.pdf]

**A**

# TgIST Bradyzoite Time Course

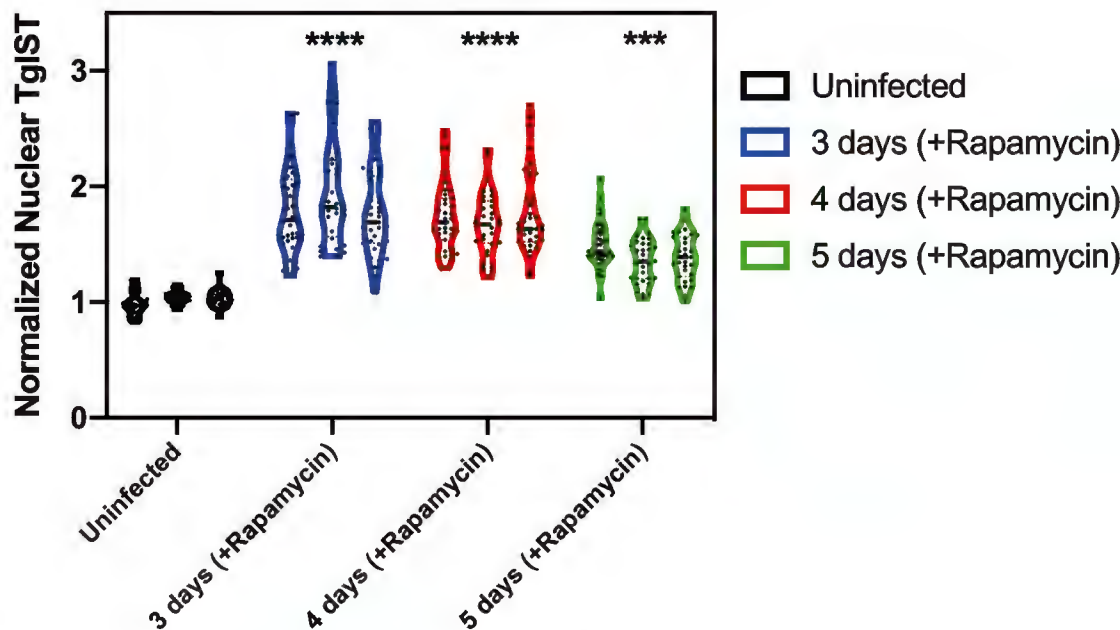**B**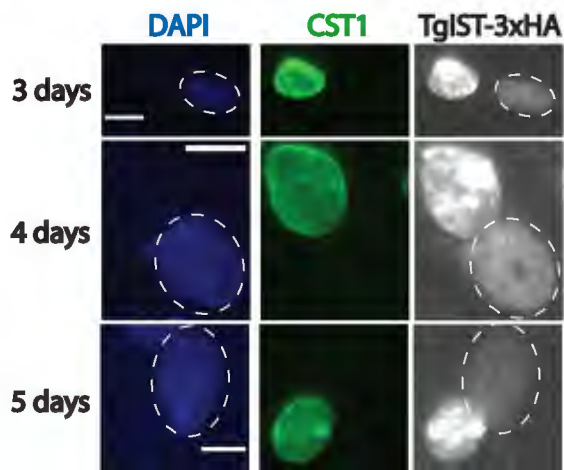

Supplement: FIG S2 [file mBio.00046-20-sf002.pdf]
