# Supplementary material for: In Vitro Characterization of Protein Effector Export in the Bradyzoite Stage of Toxoplasma gondii
Source: mBio. 2020 Mar 10;11(2):e00046-20. doi: 10.1128/mBio.00046-20 (PMC7064745; doi:10.1128/mBio.00046-20)
Supplement: TABLE S1 [file mBio.00046-20-st001.docx]

| Name | Sequence (5’ – 3’) | Purpose |
| --- | --- | --- |
| gRNA_R | AACTTGACATCCCCATTTAC | KLD sgRNA Cloning |
| KLD_MYR1_gRNA_FWD | GAAAAGCAGGAGAACGTGCCGTTTTAGAGCTAGAAATAGC | KLD sgRNA Cloning |
| KLD_GRA16_gRNA_FWD | GGTGACACGCCTCTCTCGGAGTTTTAGAGCTAGAAATAGC | KLD sgRNA Cloning |
| KLD_GRA24_gRNA_FWD | GGTTAATTACCCTTAGTGGGGTTTTAGAGCTAGAAATAGC | KLD sgRNA Cloning |
| KLD_GRA28_gRNA_FWD | GACAGATCCAGAAACTGAGAGTTTTAGAGCTAGAAATAGC | KLD sgRNA Cloning |
| KLD_TgIST_gRNA_FWD | GGCCGCCGAGTAGTGAACGGGTTTTAGAGCTAGAAATAGC | KLD gRNA Cloning |
| MYR1_CTerm_3HA_FWD | CTTTTACTACGACCGTCATTTCCGTCAGTCACATAATTCGTACCCGTACGACGTCCCGGA | C-terminal tagging |
| MYR1_CTerm_3HA_RVS | AAATTGAGTCAAAGTTGTTCGGGATTTCCCATGGCACGTTCTCCTGCGTTCTCTTCGCTATTACGCCAGC | C-terminal tagging |
| GRA16_CTerm_3HA_FWD | TTTTGACTGTTCACGCGCGAAGCGGAAAAATGATCAGATGTACCCGTACGACGTCCCGGA | C-terminal tagging |
| GRA16_CTerm_3HA_RVS | TGCCAGCACGTGGGTACCGTCCGAGAGAGGCGTGTAGCATCCTTCTGGTTAGCTCTTCGCTATTACGCCAGC | C-terminal tagging |
| GRA24_CTerm_3HA_FWD | TCCGCCGCTACGCATCGTTAAACCACCCACTAAGGGCAACTACCCGTACGACGTCCCGGA | C-terminal tagging |
| GRA24_CTerm_3HA_RVS | GCACTTTGAACACACATTCGCTGCCCCTAAAACAAACGGGCTCTTCGCTATTACGCCAGC | C-terminal tagging |
| GRA28_CTerm_3HA_FWD | TCTTCGAGCGCCGTCTCGCGGTAGCTCCAGTTATTCCGAATACCCGTACGACGTCCCGGA | C-terminal tagging |
| GRA28_CTerm_3HA_RVS | ATATGAGTGGGCTAGTGACGGCGGTTGATACCACCAATCTCTCTTCGCTATTACGCCAGC | C-terminal tagging |
| TgIST_CTerm_3HA_FWD | CGTCACCGCCATCGCTTCATCGCCGGCCGCCTAGTAGTGAACGGCGCGTCTACCCGTACGACGTCCCGGA | C-terminal tagging |
| TgIST_CTerm_3HA_RVS | CGAAACACTCAAAAATTCTGCCCGCAAATGCATCCACCCACTCTTCGCTATTACGCCAGC | C-terminal tagging |
| MYR1_CDS_FWD | CAGCAGCCACCTGAGACATA | Confirm gDNA editing |
| MYR1_3UTR_RVS | TGGACTAGGCGTCTCACGGT | Confirm gDNA editing |
| GRA16_CDS_FWD | AGTACCAGCTCAGGTGCAGC | Confirm gDNA editing |
| GRA16_3UTR_RVS | CACTGCCTCACATTTGCAGT | Confirm gDNA editing |
| GRA24_CDS_FWD | TGCGTTCTAATGACCAGGAT | Confirm gDNA editing |
| GRA24_3UTR_RVS | TGCGAACAACATACGTGCCA | Confirm gDNA editing |
| GRA28_CDS_FWD | AGCCTGCCTTGCCTGGTTCG | Confirm gDNA editing |
| GRA28_3UTR_RVS | GTTTCCCAAGACCTCGCACA | Confirm gDNA editing |
| TgIST_CDS_FWD | GTTTCAGCTCCGGGTGTGAG | Confirm gDNA editing |
| TgIST_3UTR_RVS | CATGCCCATCTTTCCGGTCG | Confirm gDNA editing |
| pLIC_Backbone_FWD_2 | GCTGGCGTAATAGCGAAGAG | TgIST-DiCre Construction |
| GA_RFPCTerm_HXGPRT_  3UTR_RVS | TAATTAAGATCTATGCATATATCGATACCGTCGACCTCGA | TgIST-DiCre Construction |
| GA_RFP_CTerm_LoxP_  YFP_FWD | TCGAGGTCGACGGTATCGATATATGCATAGATCTTAATTA | TgIST-DiCre Construction |
| GA_DiCre_HXGPRT_RVS | ATACCGTCGAGGACTAGTGGTTACTTCTCGAACTTTTTGC | TgIST-DiCre Construction |
| GA_DiCre_FWD | GCAAAAAGTTCGAGAAGTAACCACTAGTCCTCGACGGTAT | TgIST-DiCre Construction |
| GA_pLIC_DiCre_RVS | CTCTTCGCTATTACGCCAGCTCAGTCCCCATCCTCGAGCA | TgIST-DiCre Construction |
| GA_LoxP_TgIST_  5UTR_RVS | ACACCAACGCATTTCCGGGCTTCCACATAACTTCGTATAATGTATGCTATACGAAGTTATGGCCCACAGAACTGGGTCCT | TgIST-DiCre Construction |
| GA_TgIST_5UTR_  ORF_FWD | GTGGAAGCCCGGAAATGCGTTGGTGT | TgIST-DiCre Construction |
| GA_TgIST_3UTR_  DiCre_RVS | CCCGCAAATGCATCCACCCATCAGTCCCCATCCTCGAGCA | TgIST-DiCre Construction |
| GA_TgIST_3UTR_FWD | TGCTCGAGGATGGGGACTGATGGGTGGATGCATTTGCGGG | TgIST-DiCre Construction |
| GA_pLIC_TgIST_  3Arm_RVS | CTCTTCGCTATTACGCCAGCGTCGAGGGAAGGTAGTGTTT | TgIST-DiCre Construction |
| KLD_3xMyc_YFP_FWD | TCGGAGGAAGACCTCGAGCAAAAGCTTATTTCCGAAGAGGATCTTTAAATGCAGCCCACAGAAGC | TgIST-DiCre Construction |
| KLD_3xMyc_YFP_RVS | AATGAGCTTTTGCTCAAGATCCTCTTCTGAGATCAGTTTCTGTTCCTTGTACAGCTCGTCCATGC | TgIST-DiCre Construction |
| GA_FRB_Cre59_FWD | GCAAAAAGTTCGAGAAGTAAGTAATACGACTCACTATAGG | TgIST-DiCre Construction |
| GA_FRB_Cre59_RVS | ATACCGTCGAGGACTAGTGGTACCGGATCCCCCTCGGGGG | TgIST-DiCre Construction |
| GA_FRB_Cre60_FWD | CCACTAGTCCTCGACGGTAT | TgIST-DiCre Construction |
| GA_HXGPRT_RVS | TTACTTCTCGAACTTTTTGC | TgIST-DiCre Construction |

**Supplemental Table 1.** Primers used for sgRNA cloning, PCR amplification of C-terminal tagged donor sequences, confirmation of genomic DNA (gDNA) editing, and TgIST-GeneSwap-DiCre construction.

-For primers used for sgRNA cloning and C-terminal tagging with overhanging sequences, homology to the plasmid template is designated in blue.

-Mutations preventing Cas9 cleavage of donor sequences are designated in red. In coding regions, point mutations were designed to be synonymous to preserve the original encoded amino acid.
